# Challenges to Evidence Synthesis and Identification of Data Gaps in Human Biomonitoring

**DOI:** 10.3390/ijerph18062830

**Published:** 2021-03-10

**Authors:** Ana Virgolino, Osvaldo Santos, Joana Costa, Mónica Fialho, Ivo Iavicoli, Tiina Santonen, Hanna Tolonen, Evangelia Samoli, Klea Katsouyanni, Georgios Baltatzis, Flavia Ruggieri, Annalisa Abballe, Ida Petrovičová, Branislav Kolena, Miroslava Šidlovská, Carla Ancona, Ivan Eržen, Ovnair Sepai, Argelia Castaño, Marike Kolossa-Gehring, Ulrike Fiddicke

**Affiliations:** 1 Environmental Health Behaviour Lab, Instituto de Saúde Ambiental, Faculdade de Medicina da Universidade de Lisboa, 1649-028 Lisboa, Portugal; osantos@medicina.ulisboa.pt (O.S.); jfcosta@medicina.ulisboa.pt (J.C.); mfialho@medicina.ulisboa.pt (M.F.); 2 Unbreakable Idea Research, Lda, 2550-426 Painho, Portugal; 3 Department of Public Health, University of Naples Federico II, 80131 Naples, Italy; ivo.iavicoli@unina.it; 4 Finnish Institute of Occupational Health, 00032 Työterveyslaitos, Finland; tiina.santonen@ttl.fi; 5 Department of Public Health and Welfare, Finnish Institute for Health and Welfare (THL), 00300 Helsinki, Finland; hanna.tolonen@thl.fi; 6 Department of Hygiene, Epidemiology and Medical Statistics, Medical School, National and Kapodistrian University of Athens, 115 27 Athens, Greece; esamoli@med.uoa.gr (E.S.); kkatsouy@med.uoa.gr (K.K.); gbaltatz@med.uoa.gr (G.B.); 7 Department of Environment and Health, National Institute for Health, 00161 Rome, Italy; flavia.ruggieri@iss.it (F.R.); annalisa.abballe@iss.it (A.A.); 8 Department of Zoology and Anthropology, Constantine the Philosopher University in Nitra, 949 74 Nitra, Slovakia; ipetrovicova@ukf.sk (I.P.); bkolena@ukf.sk (B.K.); msidlovska@ukf.sk (M.Š.); 9 Department of Epidemiology, Local Health Authority Rome E, 00147 Rome, Italy; c.ancona@deplazio.it; 10 Public Health School, National Institute of Public Health, 1000 Ljubljana, Slovenia; ivan.erzen@nijz.si; 11 Public Health England, Harwell Campus, Chilton, Didcot OX 11 0RQ, UK; ovnair.sepai@phe.gov.uk; 12 CNSA, Instituto de Salud Carlos III, 28220 Majadahonda, Madrid, Spain; castano@isciii.es; 13 German Environment Agency, 14195 Berlin, Germany; marike.kolossa@uba.de (M.K.-G.); ulrike.fiddicke@uba.de (U.F.)

**Keywords:** HBM4EU, human biomonitoring, environmental health, data triangulation, harmonisation procedures

## Abstract

The increasing number of human biomonitoring (HBM) studies undertaken in recent decades has brought to light the need to harmonise procedures along all phases of the study, including sampling, data collection and analytical methods to allow data comparability. The first steps towards harmonisation are the identification and collation of HBM methodological information of existing studies and data gaps. Systematic literature reviews and meta-analyses have been traditionally put at the top of the hierarchy of evidence, being increasingly applied to map available evidence on health risks linked to exposure to chemicals. However, these methods mainly capture peer-reviewed articles, failing to comprehensively identify other important, unpublished sources of information that are pivotal to gather a complete map of the produced evidence in the area of HBM. Within the framework of the European Human Biomonitoring Initiative (HBM4EU) initiative—a project that joins 30 countries, 29 from Europe plus Israel, the European Environment Agency and the European Commission—a comprehensive work of data triangulation has been made to identify existing HBM studies and data gaps across countries within the consortium. The use of documentary analysis together with an up-to-date platform to fulfil this need and its implications for research and practice are discussed.

## 1. Introduction

Human biomonitoring (HBM) studies are widely used as an environmental health approach to assess human exposure to environmental and occupational chemicals, as well as to measure the internal dose of xenobiotics and/or their metabolites in human matrices, mainly in body fluids and tissues. HBM was first used to assess occupational exposure to chemicals in the early 1930s [1]; since then, HBM studies have been increasingly conducted, both as part of occupational health programs and studies monitoring the exposure of the general population to chemicals (e.g., [1,2]). Apart from the obvious relevance of HBM research [3], the cumulative number of studies sharing similar objectives but differing in sampling, data collection and analytical methods, can compromise HBM data comparability [2,4]. To tackle this major drawback, several coordinated initiatives have been set up at the European level, such as the COnsortium to Perform Human biomonitoring on a European Scale (COPHES; [4]), the DEMOnstration of a study to COordinate and Perform Human biomonitoring on a European Scale (DEMOCOPHES; [5]) and, more recently, the European Human Biomonitoring Initiative (HBM4EU; [6]).

The HBM4EU is a joint effort of 30 countries, the European Environment Agency and the European Commission, co-funded under Horizon 2020, that aims to coordinate and advance HBM in Europe. The HBM4EU work programme is designed to answer relevant open-policy questions as defined by EU services and partner countries. This includes to harmonise procedures and to produce comparable data in HBM with the ultimate goals of providing reliable evidence of citizens’ exposure to chemicals in their daily lives, as well as information on the potential health effects of the chemicals classified as “priority substances” under this project (i.e., substances to be monitored and researched by the HBM4EU consortium, at the European level). HBM4EU bridges the gap between research institutions and policy by involving scientists, chemical risk assessors and risk managers, thus ensuring that its outcomes are considered at the different stages of inception and design, implementation, and evaluation of activities developed to protect citizens’ health [6]. 

## 2. Identifying and Integrating the Best Evidence on Human Biomonitoring

Within the framework of HBM4EU, the first steps towards the harmonisation of procedures and the production of relevant, sound and comparable evidence-based knowledge that could inform different audiences are: (a) to identify and collate HBM data from existing studies and (b) to identify data gaps in this area. Specific activities in the project, within several work packages, were established to precisely answer to these needs. However, as the quantity of HBM data is growing at such fast pace, identifying and integrating all relevant evidence from different sources can be challenging. The difficulty level increases when it comes to map ongoing or concluded, not yet published, studies.

Several methods are currently available to locate and synthetise evidence (e.g., narrative and systematic literature reviews, meta-analyses, evidence mapping). All have both advantages and disadvantages, so the selection of one method over the others should be carried out on a per case basis by considering the research objective (narrow vs. broad), the effort (cost and time) required for properly conducting an evidence synthesis, or the weight of potential reporting bias to the final synthesis, among others. 

Narrative literature reviews, also known as expert reviews, depart from a broad research question and provide an overall synthesis of the current status on a given topic of interest. These are generally written by an expert in the area, who uses different sources of information without a document effort to systematically search and review the literature on a given topic. Thus, narrative literature reviews lack reproducibility with regard to the methods, mainly being a subjective evaluation of the state of the art by the writer, thus being prone to reporting bias [7,8].

Systematic literature reviews and meta-analyses, unlike narrative literature reviews, depart from a narrower research questions and follow a clear and documented search strategy, with a rigorous analysis of the included literature [7]. These two methods have been the most widely used for locating and synthesizing evidence of primary data, traditionally being put at the top of the hierarchy of scientific evidence [7,9,10]. They provide a useful summary of the state of the art on a given research and/or clinical topic, supporting informed decisions—namely, in the broad field of human healthcare [7,11]. With the increasing amount of available evidence on health risks linked to exposure to chemicals, the principles of systematic literature reviews have been increasingly applied to solve questions in the area of environmental health [12,13].

These methods, however, mainly capture peer-reviewed articles (i.e., white literature) that are mainly written in English or some other major languages, often leaving out potentially informative publications in national languages. Moreover, systematic literature reviews and meta-analyses are often unable to comprehensively identify other sources of information—namely, reports produced by regulatory agencies, data contained in dynamic databases (e.g., study registers) and on password-protected pages, conference abstracts, and academic dissertations (except for abstracts published in conference proceedings or for academic dissertations published in periodicals that are indexed in electronic search engines), among others, which are also rich sources of chemical-related evidence [14,15]. In the case of exposure data (including exposure biomarker data), for example, information available in different databases (e.g., IPCHEM [16]) are very useful because they are often not published in scientific papers (or are published with notorious delays). Conference proceedings, on another side, fall into the category of the so-called grey literature, which is not frequently searched for the purposes of literature reviews. In our opinion, this is mainly due to the following reasons: (a) searching grey literature is time consuming, since it often involves manual searching of multiple (and, often, not-so-well structured) databases; (b) grey literature is characterized by a wide heterogeneity, which makes data extraction, synthesis and integration more demanding; (c) peer review is assumed as a scientific-quality label that is not attributed to grey literature [17,18]. 

Notwithstanding, grey literature assumes a relevant role in decreasing reporting bias by also disseminating null or negative results [17,19,20], thus providing a much more complete (and comprehensive) overview of the state of the art in a given area. Moreover, curation and management of grey literature has greatly improved in recent decades [18,21]. The original concept of grey literature can be traced back to the 1920s and refers to the uncertain status of a document [22]. Back then, these documents were mainly produced for commercial purposes, not academic ones, thus lacking basic bibliographic information, such as name of the author(s) and issue of publication or page numbers, which are data required for the proper cataloguing of the documents in academic databases [18,19]. With the advent of the internet, as well as powerful index and search engines, coupled up with increased concern about data curation and management has contributed to making searches for grey literature easier [18,21,23]. Thus, and not surprisingly, the inclusion of grey literature in systematic literature reviews has been increasingly supported [17,19,24]. This source of evidence seems to be of particular relevance in the case of mapping existing studies and gaps to be addressed in the HBM field, particularly from ongoing or planned studies [15] or from non-academic organisations, in order to increase the comprehensiveness of the search. For example, well-established organisations such as the Cochrane [25], the Agency for Healthcare Research and Quality (AHRQ) [26] or the Institute of Medicine [27] recommend the search for and inclusion of grey literature and other types of unpublished data in evidence synthesis. Likewise, the European Food Safety Authority (EFSA) [28] and the International Agency for Research on Cancer (IARC) [29] emphasize that, despite their relevance, peer-reviewed documents are not necessarily rigorous, valid or transparent and that other sources of information should also be acknowledged and considered for the purposes of evidence synthesis.

Against this background, systematic literature reviews and meta-analyses tend to be considered as susceptible to reporting bias, which can lead to misleading results [30,31], thus providing an incomplete view of existing data. As a result, their quality and usefulness have been increasingly questioned [9,11,32], with alternative methods being proposed to tackle these constraints [9,13,33]. Table 1 provides a brief overview of traditional (i.e., narrative and systematic literature reviews and meta-analysis) and emerging methods for evidence synthesis (i.e., evidence mapping and next-generation systematic reviews). The latter includes the so-called next-generation systematic reviews, a group of methods with great potential, although with some limitations as well.

The discussion around the lack of transparency and poor quality of systematic literature reviews and meta-analyses has been mainly conducted with regard to evidence-based medicine, mainly due to the implications of their conclusions for routine clinical practice. With regard to environmental health, and more specifically concerning evidence synthesis on human health effects of exposure to chemicals, multiple sources of information, including HBM data, experimental studies, among others, should be considered. This makes environmental health data *lato sensu* fragmented and scattered and adds additional complexity to the activity of summarizing and integrating data from projects at any stage of execution. Moreover, the interest in assessing human exposure to chemicals goes beyond a merely academic one. Instead, data on exposure and a chemical’s fate in human matrixes can be, if available, used to support risk assessment [38] and risk management actions, which are now a part of routinely conducted national health surveys in some countries, including Germany and the USA [1].

A common practice to minimise publication bias when conducting a systematic literature review or a meta-analysis is to obtain unpublished data [39]. Methods that are usually considered to be at the bottom of hierarchy of evidence can be pivotal in identifying relevant evidence when other data are unavailable or insufficient [40]. For example, the contact with the researchers who are responsible for the studies can be a suitable option to complement data obtained through systematic literature reviews, while addressing the concerns regarding the credibility of empirical findings of reported data in the scientific literature. Study registries provide an alternative form of interaction with the researchers of a given project, thus representing a powerful source of published and unpublished data. Study registries can, however, be linked to several preconceptions that may hamper adherence to data reporting, namely: (a) technological requirements of online platforms can be seen as quite challenging and difficult to use/manage; (b) the lack of integration with other electronic datasets may demand additional time investment in filling in the same data over and over again, which can be regarded as an unnecessary burden; (c) data registration can be thought of as only allowed to large-scale studies and not to local ones.

## 3. Data Triangulation: The Case of HBM4EU

In order to overcome the obstacles to gather a comprehensive list of concluded, ongoing and planned HBM studies under the HBM4EU initiative, data triangulation [34] is proposed to identify available HBM projects’ methodological information and data gaps across European countries within the consortium—namely, through the complementary use of (a) systematic literature reviews (i.e., documentary analysis), covering peer-reviewed papers and HBM-related documents such as conference proceedings, academic dissertations and reports, and (b) an online, permanently open, HBM research projects register platform. These mapping approaches were selected on the bases of their potential, if used in combination, to maximize the coverage of scattered data. The strategy adopted within the HBM4EU was based on the following assumptions: (a) systematic literature reviews are vulnerable to bias and confounders inherent to the results reported in peer-reviewed papers; (b) despite reporting guidelines being currently widely used for empirical data, such as CONSORT [41] and STROBE [42], and PRISMA [43], for systematic reviews, this was not the case a few years ago; even now, if not mandatory by the journal, their use is often a choice of the author, impairing the quality of systematic reviews when these were not employed; (c) the producers and target audience of HBM studies are much broader than academic researchers only, also including regulatory organisations, health authorities, stakeholders, among others; (d) findings of ongoing studies, especially those running for a long period of time (or, for any reason, interrupted), and the protocol of planned studies, are unlikely to be already published. 

The first efforts towards this identification process started in 2017 with literature reviews targeting HBM studies and human exposure to the chemical substances. In parallel, a web-based questionnaire was developed and disseminated within the HBM4EU consortium to identify concluded, ongoing or planned HBM studies that could inform project activities. The questionnaire provides detailed information about each study, covering: (i) a general characterisation of the study, (ii) information on target population and methods for selecting participants, (iii) fieldwork details, (iv) collected indicators, (v) adopted quality control procedures, (vi) protection, availability and conditions of access and use of data (and of biological samples), (vii) communication strategies, and (viii) difficulties encountered along the study. Details about the literature search, the construction and contents of the electronic questionnaire can be found in Virgolino et al.’s study [44]. Briefly, 92 studies were reported in the questionnaire, while 22 and 58 different projects were found using literature reviews and reviews of HBM books of proceedings, respectively. Only 18 studies were mapped simultaneously by the different search sources [44].

The questionnaire was then updated/adapted based on the lessons learned from the received answers and on the use of the data, and evolved into an online platform accessible to partners within the HBM4EU consortium for access to the information about data sources, search, visualisation and reporting [45]. An overview of the platform dimensions and corresponding variables, as well as its main features, is provided in Table 2. This work, conducted to fulfil HBM4EU’s objectives, represents a first step towards data harmonisation at the European level. Moreover, data gathered, and the procedures generated can be used in future developments in the HBM area.

## 4. Implications of Harmonised Procedures for Human Biomonitoring

As previously mentioned, the number of HBM studies has been vertiginously increasing, thus leading to a steeping rise in the number of peer-reviewed publications describing HBM approaches to assess human exposure to chemicals, but also of experimental toxicological studies. However, HBM studies are highly heterogeneous regarding the procedures adopted, even at the substance level—human (and experimental animals) matrixes sampled (mostly blood and urine, but with an increasing variety of biological materials) and methods for substances’ quantification differ between studies. This constitutes a serious challenge to comparisons across studies and, ultimately, to the identification of potential causality associations between exposure and disease (for a discussion on the relevance of data integration for causality, see [46]). The lack of harmonised procedures in the health sciences has motivated much discussion and numerous efforts towards retrospective (e.g., Maelstrom Research Platform; [47]) and prospective (e.g., COPHES; [4]) data harmonisation have been conducted. Whereas the latter involves the adoption of identical procedures among studies, retrospective harmonisation is more challenging as it involves the assessment of existing data in terms of comparability among studies [48]. Concerning HBM in Europe, methodological heterogeneity among studies is a major challenge that precludes a comprehensive assessment of the exposure to chemicals and potential health effects to European citizens. Moreover, the General Data Protection Regulation can be a hurdle for joint analysis of data, which might be even more critical in large-scale studies. 

From a different angle, more upstream, the enormous number of studies in HBM makes it difficult to map them all in the first place. Large-scale studies usually get funded and are disseminated and, therefore, these are more easily located using any of the abovementioned methods. This brings an additional challenge as one study can be identified more than once but not recognized as such, contributing to a biased estimation of the total number of HBM projects. The use of a strategy similar to that of capture–recapture models, which have their origins for estimates of animal abundance, might be a valuable strategy allowing the estimation of the true size of existing studies in the area. This technique evaluates the degree of overlap among different lists of studies identified from existing data sources [49,50].

In this context, HBM4EU assumes a pivotal role to tackle heterogeneity among studies and some progress has already been made—namely, regarding the identification of priority substances (for details, see [51]) and the alignment of HBM studies across Europe (for details, see [52]). However, this would not have been possible unless relevant studies were identified. Mapping HBM initiatives at any stage of development and gathering information about the methods, target population, substance(s) of interest and geographical area where the study was conducted, among other details, not only provides a good summary of the evidence available, but also allows the identification of knowledge gaps, such as research design approach, such as geographical and temporal ones. This information is extremely useful as it can assist the development of health and wellbeing policies—namely, by allowing the identification of priorities for risk assessment and intervention. 

Collaborative research involving multiple research and governmental centres at the national and international levels is critical for advancing health sciences. This is the perfect setting for design comparable studies in a prospective manner. An added value would be, for example, that data from several studies could be pooled together, providing more power for statistical analysis than individual studies would have. On the other hand, large consortiums, such as the HBM4EU, profit from the expertise of researchers and regulators to identify and collate relevant data (both retrospectively and prospectively), harmonise procedures in HBM and ensure that the databases are up to date. This is also the right forum for knowledge exchange, identification of research and action priorities, formulation of new questions to be addressed, and establishment of new collaborations. 

## 5. Conclusions

There is no gold standard for integrating the best research evidence. Several methods have been proposed to map both published and unpublished materials in order to gather a comprehensive overview of the existing evidence on a given area. Within the HBM4EU initiative, efforts have been made to establish a solid background knowledge of scientific advances in the HBM field, through the creation of an online platform with a collection of concluded, ongoing or planned studies of the consortium partners, complemented by a thorough review of the available literature on HBM, as a first step towards the development of harmonisation of procedures. The next steps under this realm include the improvement and continuous update of the online platform already created, also stimulating a snowball procedure for the identification and invitation of principal investigators of small-scale though relevant HBM projects. By continuously making an effort to have a broad picture of what is being carried out in the HBM area, this platform may be a useful tool for other research initiatives, even beyond HBM—namely, in the areas of clinical research.

## Figures and Tables

**Table 1:** Overview of methods for evidence synthesis of primary data.

Method	Main Defining Features	Pros	Cons
Narrative literature review [7,8]	Subjective evidence synthesis	▪ Can cover grey literature ▪ Normally, in-depth reflection about the subjects under study, pointing out to possible research, clinical or policy avenues ▪ Hypothesis generating ▪ Valuable basis for theory-developing processes	▪ Broad research question ▪ No systematic, reproducible search strategy ▪ No quality assessment of the included studies ▪ Qualitative analysis of the findings reported in the selected papers ▪ Reporting bias
Systematic literature review [7]	Objective evidence synthesis (includes narrative and interpretative synthesis, with methodological evaluation of the identified studies)	▪ Narrow research question ▪ Clear search strategy ▪ Studies selected based on predefined inclusion/exclusion criteria ▪ Study quality assessment included ▪ Quantitative and/or descriptive analysis of the findings reported in the papers	▪ Reporting bias ▪ Grey literature is not usually searched
Meta-analysis [34,35]	Statistical analysis of outcome indicators (e.g., means, odds ratio) from studies identified after a systematic literature search	▪ Narrow research question; clear search strategy ▪ Studies selected based on predefined inclusion/exclusion criteria ▪ Quality assessment of the studies included ▪ Provides a pooled effect ▪ Allows for the proposal of new hypotheses	▪ Reporting bias ▪ Grey literature is not usually searched ▪ Highly dependent of the methodological homogeneity of the research in a specific field
Evidence mapping [13,33]	Queryable database of evidence	▪ Allows the identification of evidence trends and gaps in a research area ▪ Makes use of graphical/visual tools and/or interactive, online databases	▪ Broad research objectives ▪ Scoping, methods and reporting guidelines are not clear yet ▪ No synthesis of the findings provided in the studies included ▪ Critical appraisal of the quality of the studies included is not mandatory ▪ Regular update of the database is challenging
Next-generation systematic reviews [9,36,37]:			
▪ Prospective meta-analysis	Meta-analysis of data from multiple studies/cohorts that were designed to be combined when completed	▪ Hypotheses specified a priori, before knowing the results of individual studies/cohorts ▪ Selection criteria applied prospectively ▪ A priori definition of intended analyses, allowing potential dependence on unreliable data on specific subgroups	▪ Broad research objectives ▪ Requires the coordination of multiple stakeholders
▪ Individual level meta-analysis	Meta-analysis of raw data from multiple studies	▪ Direct work with original research data ▪ Improved data quality ▪ Production of more reliable results	▪ Requires data cleaning and the harmonisation of variables across studies ▪ Raw data are hard to obtain (with relevant ethical and data protection challenges) ▪ Requires dedicated staff with different skills ▪ Can take longer and be more expensive than other meta-analyses
▪ Network meta-analysis	Meta-analysis of data from multiple (network of) studies on a given health condition	▪ Use of direct and indirect evidence ▪ More precise estimates of intervention effects ▪ Allows for the estimation of the hierarchy of interventions	▪ Broad research question ▪ Builds on direct and indirect evidence to estimate the relative effect of each treatment/intervention under study which can originate incoherence of results

Note: Umbrella reviews (systematic literature reviews of systematic literature reviews) are another example of next-generation systematic reviews but are out of the scope of this work.

**Table 2:** European Human Biomonitoring Initiative (HBM4EU) platform: main objectives, dimensions and variables, and overall features.

**Objectives**	To integrate data on concluded, ongoing and planned studies on HBM To identify data gaps and needs in HBM research conducted in Europe
**Dimensions and variables**	General characterisation of the study: Overall information on the study, including name and acronym, principal investigator, responsible institution, contacts, type of study, implementation level, country and language of data collection, starting and ending dates, budget and funding institutions, and ethical approval. Target population and method for selecting participants: Information on study design, study setting, target groups (age and sample size), inclusion and exclusion criteria, control group, sampling, recruitment and consent procedures. Fieldwork: Information on period of data collection, questionnaire(s) used, groups of substances under study, collected indicators. Collected data: Information on collected indicators. Quality control procedures: Information on preanalytical quality assurance/quality control, internal quality control procedures, standard operating procedures, accreditation of the laboratory and other certifications. Data protection, availability and conditions of access and use: Information on data storage and access. Communication: Information on the dissemination of the study to public authorities, study participants, health institutions, scientific community and the general public. Obstacles, shortcomings and difficulties: Information on overall constraints and difficulties, including participants’ recruitment and data collection.
**Features**	Data access: Different levels of access for registered and unregistered users; Brief or detailed information on the reported studies, according to users’ access levels. Data search: Study search by different queries: name of the study, status, country of data collection, chemical substance under study and biological samples analysed; Exportation of the search results. Data visualisation and reporting: Summary of the main statistical indicators of the studies included in the platform.

## Data Availability

No new data were created or analyzed in this study. Data sharing is not applicable to this article.
